# Like a Punch in the Gut: A Novel Perspective On Annual Recurrences of Ulcerative Colitis

**DOI:** 10.1093/crocol/otae050

**Published:** 2024-10-17

**Authors:** Sasha Johnston, Aileen Fraser, Carrie Biddle, Jennifer Wild

**Affiliations:** Department of Experimental Psychology, University of Oxford, Oxford, UK; National Health Service England, Workforce Training & Education Directorate, Plymouth,, UK; Department of Gastroenterology, University Hospitals Bristol and Weston Trust, Bristol, UK; National Health Service England, Workforce Training & Education Directorate, Plymouth,, UK; Department of Experimental Psychology, University of Oxford, Oxford, UK

## Abstract

**Background:**

Ulcerative colitis (UC), a chronic inflammatory bowel disease, causes stomach pain, diarrhea, and rectal bleeding. The exact cause is unknown, but it is thought to involve genetic, environmental, and psychological factors. Some people experience annual flare-ups without obvious reason. This article adopts a theory-driven approach to consider how and why past traumatic events may contribute to annual flare-ups.

**Methods:**

We applied learning theory, which explains the development of re-experiencing phenomena in post-traumatic stress disorder (PTSD), to better understand the occurrence of annual flares in patients living with UC.

**Results:**

Two possibilities emerged in which associative learning may contribute to annual UC flares. First, flare-ups could be a physical response to sensory cues in the present that overlap with trauma experienced at the first onset of UC. Annual episodes may strengthen the UC flare as a learned physiological response to trauma reminders. Second, flare-ups may result from elevated stress due to trauma re-experiencing at anniversaries. Sensory features of the initial UC trauma may be associated with strong reactions, which generalize to similar stimuli, triggering re-experiencing symptoms and increasing psychological stress. Elevated stress raises glucocorticoid levels, promoting UC-specific inflammation. Stimulus discrimination from cognitive therapy for PTSD may help to over-ride the associations that have formed between sensory features of past trauma, linked reactions, and similar cues in the present.

**Conclusions:**

Research is needed to understand how traumatic events influence the onset and recurrence of ulcerative colitis, as well as the potential benefits of stimulus discrimination for reducing the frequency of annual flares.

## Graphical Abstract

**Graphical Abstract:**
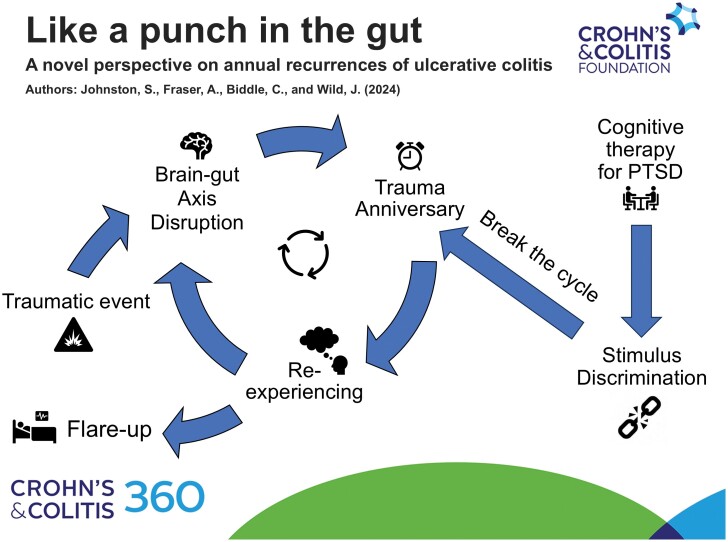

## Introduction

Ulcerative colitis (UC) is a chronic inflammatory bowel disease (IBD) that affects the mucosa of the colon and rectum. It is estimated that IBD affects more than 0.3% of the global population and about 146 000 people in the United Kingdom have UC, with a prevalence of 243 per 100 000 population.^1-5^ The clinical course of UC is characterized by periods of remission and relapse, with symptoms ranging from mild to severe. Symptoms vary from person to person, but the most common symptoms are loose, urgent bowel movements, abdominal pain, fatigue, blood and mucous in the stool, weight loss, and anemia. These symptoms can impair the quality of life and psychological and physical well-being of patients.^6^

The exact causes of UC are unknown, but it is thought to result from a complex interplay of genetic, environmental, and psychological factors.^7^ Genetic factors are linked to an increased risk of developing UC, with more than 200 susceptibility loci identified.^8^ However, due to its complex pathophysiology, IBD cannot be explained by a few genes or pathways alone. Environmental and lifestyle factors are thought to be important and include smoking, diet, infections, medications, and stress which may influence the gut microbiota, the immune system, and the intestinal barrier function. Psychological factors, such as anxiety, depression, and trauma, may also affect the onset and course of UC, through the modulation of the brain-gut axis.^9^

The brain-gut axis is a bidirectional communication system that links the central nervous system (CNS) and the enteric nervous system of the gastrointestinal tract. The brain-gut axis involves neural, hormonal, and immunological pathways, and is influenced by the gut microbiota, which is the collection of microorganisms that inhabit the gut.^10^ The brain-gut axis plays a key role in the regulation of gut motility, secretion, immunity, and sensation, as well as in the modulation of mood, cognition, and behavior.^11^

A number of “classic” extraintestinal manifestations of UC, where inflammatory pathology arises from distant sites in the body, typically involve inflammation outside the gastrointestinal tract, such as anemia and arthritis. However, the mind-body connection is an important factor in extraintestinal manifestations of UC, as the connection between psychological stress or psychological sequala following traumatic events and IBD flares is well-documented.

## Psychological Stress

There is growing evidence that psychological factors such as stress, depression, anxiety, and traumatic events may influence the onset, course, and outcome of chronic diseases such as UC.^12^ Psychological stress can disrupt the brain-gut axis, leading to alterations in the gut microbiota composition and diversity, causing increased intestinal permeability, enhanced immune activation, and altered visceral sensitivity.^13^ These changes can contribute to the development and exacerbation of UC, as well as to the occurrence of extraintestinal manifestations, such as joint pain, skin lesions, and eye inflammation. Conversely, UC can also affect the brain-gut axis, causing changes in the CNS structure and function, such as reduced hippocampal volume, impaired memory, and increased anxiety.^14^

Traumatic events, such as actual or threatened death, serious injury, or sexual violence, may play a unique role in the onset and recurrence of UC.^15^ Examples of traumatic events known to be associated with long-term psychological harm include natural disasters, wars, the sudden death of a loved one, accidents, assaults, abuse, or neglect. An individual’s reaction to a traumatic event can have lasting effects on their mental and physical health and are associated with post-traumatic stress disorder (PTSD), depression, anxiety, substance abuse, chronic pain, and cardiovascular diseases.

Trauma may occur around the first onset of UC or the onset of the illness itself may be experienced as a traumatic event if the individual perceived they were at risk of death or serious harm at the time. Such events may be associated with intrusive re-experiencing. When traumatic events are re-experienced, such as in the form of intrusive memories or strong emotions in response to reminders of the trauma, they may influence the perception of pain and the coping strategies people utilize to deal with symptoms, which in turn may lead to greater distress and disability.

In the context of UC, some patients report annual flare-ups of their condition, without obvious triggers, such as infection, dietary changes, or medication non-adherence. These flare-ups can occur around the same time of year and may be associated with seasonal variations in temperature, sunlight, viruses, or allergens.^16^ However, another possible explanation for annual flare-ups is that they may be a physiological response, a re-experiencing symptom linked to the original index trauma temporally associated with the first onset of UC. This could be conceptualized as similar to physiological responding to a trauma reminder, commonly seen in PTSD, yet without experiencing the full range of PTSD symptoms required for diagnosis. Or, the UC flare may be the result of trauma re-experiencing at anniversaries, which elevates psychological stress, glucocorticoids, and inflammation.

The accumulation of stressors is known to lead to flare-ups.^17–19^ Traumatic experiences can cause some individuals to become highly reactive to and easily triggered by a range of stressors, and not just those that remind them of the index traumatic event. As such, the accumulation of everyday stressors can activate the body’s physiological stress response, especially during the lead-up to and the time of the anniversary of a traumatic event, causing chronic inflammation which is known to play a role in UC flares. The bi-directionality of the brain-gut axis can increase the inflammatory response of the CNS, which in turn contributes to psychological stress and IBD activity.^20^

The re-experiencing symptom cluster for PTSD in the Diagnostic and Statistical Manual of Mental Disorders (5th edition; DSM-5) and International Classification of Diseases (11th edition; ICD-11) include physiological responses to trauma reminders as a potential symptom. In the context of PTSD, physiological responding is typically experienced as heart palpitations or sweating and activation of the sympathetic nervous system in response to trauma reminders. Patients are often unaware of the trauma triggers which bring about these reactions and there is a long-standing literature on anniversary reactions to trauma,^21,22^ such that trauma-focused cognitive behavioral therapy for PTSD (TF-CBT), recommended by the National Institute for Health and Care Excellence (NICE), includes a relapse plan at the end of treatment for PTSD, focusing on how to approach anniversaries of trauma.

What we are proposing here is that re-experiencing phenomena commonly associated with trauma may be relevant to conceptualizing annual UC flares. First, it is plausible that annual UC flares could be a physiological response to sensory cues in the present that overlap with trauma experienced at the first onset of UC. Second, the UC flare may, in response to trauma reminders at anniversaries, be the result of a triggered psychological response, such as a distressing memory, which elevates stress. Associative learning is relevant to these processes. Annual episodes may strengthen the UC flare as a learned physiological response to trauma reminders. It is equally plausible that sensory features of the trauma experienced at first onset UC may be associated with strong reactions, which generalize to similar stimuli, triggering re-experiencing symptoms, and elevating psychological stress. Schneider et al.^23^ demonstrated that psychological stress elevates glucocorticoid levels and drives the generation of an inflammatory subset of enteric glia promoting inflammation specific to UC.

From a theoretical perspective, conceptualizing UC flares as a re-experiencing phenomenon fits with associative learning. Sensory cues in the present that overlap with sensory features of the trauma, such as similar sounds, colors, smells, shapes, movements, or bodily sensations, can trigger physiological and psychological reeexperiencing. During trauma, the brain is attuned to perceptual features of the experience (data-driven processing), leading to strong perceptual priming, which lowers the threshold for spotting similar perceptual patterns in the environment. Through learned associations, the stimuli become associated with strong affective and physiological responses, which can be generalized to other similar stimuli. In line with the role of associative learning, re-experiencing includes strong affective and physiological responses that are clearly related to the trauma, without the person recognizing that a trauma memory has been triggered (affect without recollection).^24^

The involuntary re-experiencing of the traumatic event is triggered by a wide range of stimuli and situations. Many of the trigger stimuli are cues that do not have a strong semantic relationship to the event but instead are simply cues that are temporally associated with the event. Common examples are physical cues similar to those present shortly before or during the traumatic event (eg, the shape of a person, spatial cues, smells, a pattern of light, and particular phrases said in a certain tone of voice), similar emotional states (eg, feeling helpless or trapped) or other similar internal cues (eg, touch on a certain part of the body, proprioceptive feedback from one’s own movements or posture).^25^

Therefore, it is plausible that stimuli in the present related to a traumatic event that occurred around the same time of year as the onset of UC may precipitate flare-ups. Through associative learning, such stimuli may trigger physiological responses at anniversaries. This physiological response would include activation of the brain-gut axis and a state of chronic inflammation and stress experienced as a UC flare.^26^ Additionally, anniversaries may trigger memory of the index trauma and affective recollections more strongly than at other time periods, increasing psychological stress, elevating glucocorticoid levels, and generating an inflammatory subset of enteric glia promoting inflammation specific to UC.

## Anniversary Reactions

Anniversary reactions are a common psychological phenomenon among individuals who have experienced trauma.^27^ If the triggering event was a public event (such as a natural disaster or terrorist event), media attention received during anniversaries can intensify reactions.^28^ Anticipating the anniversary can create distress as individuals may wonder why they are still affected by the event.^29^ As the anniversary approaches physical manifestations of anniversary reactions can occur, such as re-experiencing, where individuals may relive the feelings, bodily responses, and thoughts they had during the traumatic event. Some may experience increased emotional intensity with symptoms that include anger, despair, resentment, irritability, and sorrow. Difficulty sleeping, dreams, flashbacks, and changes in appetite can also occur.^30^

Current research indicates that chronic glucocorticoid signaling drives the effect of stress on IBD. Glucocorticoid receptors are found in most neurons and glial cells in the brain. Glial cells communicate stress signals from the CNS to the semi-autonomous enteric nervous system within the gastrointestinal tract. Chronically elevated levels of glucocorticoids drive the generation of an inflammatory subset of enteric glia that promotes inflammation seen in UC and Crohn’s Disease (IBD).^23^

Glucocorticoid receptor sensitivity is implicated in post-traumatic stress reactions. Anniversary reaction, as an underlying cause of the annual prevalence of UC, is supported by case reports and observational studies which have reported a temporal association between traumatic events and UC onset or exacerbation.^30,31^ The UC flare may represent a physiological response to sensory cues in the present that share features with the past trauma. Or, the UC flare may be the result of a triggered psychological response to trauma reminders, such as distressing memory, elevating stress, and glucocorticoids, leading to inflammation. Anniversaries will share many overlapping features with a past traumatic event associated with first onset UC.

Traumatic events can disrupt the brain-gut axis and cause dysbiosis, which is an imbalance in the composition and function of the gut microbiota. Dysbiosis may impair the gut barrier function and increase the permeability of the gut wall, allowing the translocation of bacteria and toxins into the bloodstream. This may trigger a systemic inflammatory response, characterized by the activation of immune cells and the release of pro-inflammatory cytokines. Inflammation may also affect brain function and behavior, creating a vicious cycle of stress, dysbiosis, and inflammation.

The body may remember and experience inflammatory processes even after the memory of the event fades, due to the epigenetic (eg, methylation and acetylation) and neuroplastic changes (eg, alterations in the structure and function of neurons and synapses, such as dendritic branching and synaptic pruning).^32^ This may affect the regulation of immune and inflammatory genes, making them more or less responsive to stimuli, and may affect the connectivity and activity of brain regions involved in fear memory and emotion processing. These epigenetic and neuroplastic changes may persist for a long time, even after the traumatic event is no longer present or recalled, and can result in chronic glucocorticoid signaling.

## Treatment Options

There is no known cure for UC and current treatment options for UC include medications, such as aminosalicylates, corticosteroids, immunosuppressants, and biologics, which aim to reduce inflammation and maintain remission.^33^ Surgery, such as colectomy or ileoanal pouch anastomosis, may be considered for patients who have severe or refractory disease, or complications such as perforation, or cancer.^34^ However, these treatments may not be effective or well-tolerated by all patients and can have side effects, such as infections, osteoporosis, or pouchitis.

This leads us to consider whether the best treatment and support options are being offered to UC patients. If the anniversary reaction results in flare-ups, then addressing the underpinning psychological sequelae may reduce UC reoccurrence and induce remission. For patients who have trauma-related UC flares, there is potential for evidence-based psychological intervention to be beneficial. While psychological support is recommended by IBD UK standards, the need is poorly met by IBD services.^35^ Evidence for the effectiveness of psychological interventions in the management of psychological sequelae caused by UC is weak and this may relate to the type of intervention offered, which may not target the psychological mechanism causing the flare-ups. ^36^

We have outlined a theoretically derived explanation for UC flare-ups drawing on a robust psychological model of the reexperiencing symptoms that can develop following trauma exposure. Utilizing components of evidence-based intervention informed by this model, such as stimulus discrimination, may offer a promising strategy for patients who experience anniversary-related UC flares.^37^ Stimulus discrimination is a trauma treatment tool used in cognitive therapy for PTSD, which aims to help patients discriminate between the sensory features in the present that overlap with their index trauma. In this case, the index trauma is the trauma temporally related to first onset UC and would refer to a traumatic event, such as the sudden death of a loved one or threatened violence, experienced at the time. The onset of UC itself could be considered to be a traumatic event if it was experienced with fear of death or serious harm.

Cognitive therapy for PTSD (CT-PTSD) is a NICE-recommended first-line treatment for the disorder that demonstrates high rates of recovery in randomized controlled trials and effectiveness studies.^38–43^ CT-PTSD is based on a robust cognitive model^25^ that identifies three core processes that cause PTSD to persist, each one reinforcing a sense of threat in the present. The first process refers to the disjointed nature of trauma memories, which are easily triggered and poorly integrated with an individual’s autobiographical memories so that they are retrieved without a context as if the event was happening now. The second process relates to the meanings the patient makes of their trauma and what has happened since, captured in their idiosyncratic appraisals of the trauma, its worst moments, and subsequent events. The third process refers to the strategies the patient may engage in to cope with their difficult symptoms, feelings, and memories yet which typically maintain them, such as pushing memories out of mind, dwelling on parts of the trauma, and over-checking for danger.^44^

CT-PTSD targets the processes that cause PTSD to persist. The treatment has three aims: (1) to elaborate and update the trauma memory in order to reduce reexperiencing symptoms, (2) to modify negative appraisals, and (3) to change strategies that maintain the patient’s sense of threat while at the same time helping the patient to reclaim or rebuild activities in their life that provide a sense of worth and meaning. The treatment typically consists of 10 to 12 sessions.

Latent growth curve analyses demonstrate that the processes specified in the cognitive model of PTSD^25^ account for PTSD symptom reduction. That is, changes in cognitive processes, such as trauma-related appraisals and unhelpful responses to unwanted memories (ie, rumination, avoidance, and numbing), precede PTSD symptom change with treatment.^45–47^ The treatment is available in a therapist-assisted digitally enabled format, also associated with high rates of recovery,^48^ which may be ideally suited to UC patients who, at the time of flare-ups, may be bedridden.

It is unlikely that UC patients with anniversary-related flare-ups would need an entire course of CT-PTSD. This is, of course, an empirical question. We propose that conceptualizing the anniversary as a psychological response to the index trauma informs the use of evidence-based strategies for reducing the likelihood of a flare-up. Stimulus discrimination drawn from CT-PTSD may help to reduce the incidence of flare-ups for UC patients who suffer from anniversary reactions Patients learn to spot how a trigger in the present is similar to the past trauma and then focus their attention on all the ways the trigger is different to the past event. It can be useful to intentionally enhance the perceptual differences. For example, if a patient’s trauma was threatened violence and they were trapped in their home at the time, moving about when they are reminded of their trauma could help to enhance perceptual differences, physically demonstrating that they are no longer trapped.

While interventions, such as antidepressant medication, gut-directed hypnotherapy, and mindfulness-based stress reduction, have shown promising results in clinical trials and meta-analyses,^49–51^ more research is needed to confirm their efficacy and safety and to identify when and how they work. Psychodynamic psychotherapy may offer an alternative approach, guiding patients to make links between trauma that may have occurred around the onset of UC illness and anniversary symptoms. However, while insight is beneficial, it is unlikely to break the associations that have formed between sensory features of past trauma, linked reactions, and the generalization to similar cues in the present. We see treatment tools drawn from cognitive therapy for PTSD as a potentially more promising approach since CT-PTSD targets the cognitive processes hypothesized to give rise to the flare-ups. Stimulus discrimination may be of particular value since it is a practical method for reducing re-experiencing phenomena.^45–47^

There is a need to understand more about the relationship between psychological well-being and gut health. Personalized care is one of the five major changes recommended in the NHS Long Term Plan^52^; giving people choice and control over the way their care is planned and delivered based on ‘what matters’ to them and their individual strengths and needs. A personalized care approach acknowledges that needs arise from circumstances beyond the purely medical, and connects people to care and support options available in their communities. Aligning with these principles, personalized IBD care could be improved by encouraging IBD patients to share relevant history and to journal and track symptoms over time. This information would help to inform personalized care plans and target interventions to prevent future flare-ups.

## Conclusion

Learning theory, which explains re-experiencing phenomena associated with traumatic events, may be relevant to understanding the phenomenon of annual flares among patients living with UC. Sensory cues during anniversaries that overlap with sensory features of a traumatic event linked to first onset UC may trigger physiological re-experiencing in the form of an annual flare-up. The annual flare-up is conceptualized as a learned physiological response to cues in the present that match the past traumatic event. With equal plausibility, sensory features of trauma experienced at first onset UC may be associated with strong reactions, which generalize to similar stimuli. Such stimuli are plentiful at anniversaries and may trigger post-trauma reexperiencing symptoms, such as distressing memories or strong affect, which elevates psychological stress, glucocorticoids and in turn, inflammation.

Personalized IBD care plans that include psychological interventions may be beneficial for patients with trauma-related UC flares; drawing on evidence-based intervention for PTSD, such as cognitive therapy for PTSD, which could help to reduce the risk of reexperiencing symptoms triggering UC flares. Further research is needed to elucidate the role of traumatic events in UC pathogenesis and management and to evaluate the efficacy and feasibility of relevant components of evidence-based trauma treatment for PTSD for UC patients.

## Data Availability

No new data were created or analyzed for this manuscript.
